# Robot-Assisted Heller Myotomy Versus Laparoscopic Heller Myotomy: A Systematic Review and Meta-Analysis

**DOI:** 10.7759/cureus.48495

**Published:** 2023-11-08

**Authors:** Karim Ataya, Ayman Bsat, Almoutuz Aljaafreh, Hussein Bourji, Amir Rabih Al Ayoubi, Najwa Hassan

**Affiliations:** 1 Institute of Minimally Invasive Surgery, King’s College Hospital, London, GBR; 2 Department of General Surgery, American University of Beirut Medical Center, Beirut, LBN; 3 Upper Gastrointestinal Surgery, King’s College Hospital, London, GBR; 4 General Surgery, University of Pittsburgh Medical Center, Pittsburgh, USA; 5 General Medicine, Faculty of Medical Sciences, Lebanese University, Beirut, LBN; 6 Department of Surgery, Beirut Arab University, Beirut, LBN

**Keywords:** esophageal myotomy, general and laparoscopic surgery, robotic-assisted surgery, achalasia cardia, heller myotomy

## Abstract

Robot-assisted Heller myotomy (RAHM) is an increasingly popular alternative to the traditional laparoscopic Heller myotomy (LHM) in the surgical management of achalasia, with similar outcomes and potentially lower complication rates. We aimed to systematically review the literature by comparing the technical success, outcomes, and complications of RAHM and LHM.

We searched PubMed, Medline, and Cochrane Central Register for articles published between 2001 and 2023. Data on technical success, clinical outcomes, length of hospital stay, esophageal perforation rate, and overall mortality were extracted.

A total of 11 articles were included in the study, comparing a total of 3,543 RAHM and 15,434 LHM cases. The mean operative time was significantly higher in the RAHM procedure with a total mean difference of 23.95 (95% confidence interval (Cl) 17.09, 30.81; p < 0.00001; I^2^ = 99%). However, the RAHM was associated with a significantly shorter hospital stay, with a total mean difference of -0.24 (95% Cl = -0.40, -0.08; p < 0.00001; I^2^ = 81%). The volume of blood loss was significantly smaller in RAHM with a total mean difference of -61.11 (95% CI = -150.31, 28.09; p < 0.00001; I^2^ = 99%). Esophageal mucosal perforation was significantly lower in RAHM with an odds ratio of 0.36 (95% CI = 0.16, 0.82; p = 0.02; I^2^ = 22%). Both procedures were associated with similar rates of symptom relief. Although no mortality was recorded in patients who underwent RAHM as opposed to 16 cases in patients who underwent LHM, no statistically significant difference could be reached.

Our results demonstrate that while both procedures yield comparable clinical outcomes, RAHM is associated with a lower overall complication rate, particularly a lower rate of esophageal mucosal perforation, shorter hospital stay, and possibly a lower mortality rate. This confirms that RAHM is a viable and justifiable alternative to the conventional LHM in the surgical management of achalasia.

## Introduction and background

Achalasia, a disorder affecting esophageal smooth muscle motility, is a relatively uncommon pathology due to the failure of relaxation of the lower esophageal sphincter and the absence of peristalsis in the esophagus, leading to functional obstruction at the gastroesophageal junction [1]. Current therapeutic methodologies for primary idiopathic achalasia include nonsurgical or surgical interventions. Nonsurgical alternatives encompass pharmacotherapy, endoscopic botulinum toxin injection, or pneumatic dilatation [2]. Conversely, interventional alternatives include laparoscopic or robot-assisted Heller myotomy (LHM or RAHM) and peroral endoscopic myotomy [2,3].

The first case of RAHM was reported in 2001 [4]. Since then, this procedure has risen in popularity as an evolution of the gold standard for achalasia surgery, the LHM. This adoption has accelerated in the past decade as evidence showing the enhanced three-dimensional visualization of the robotic system and the increased degrees of motion of the robotic arms led to a more technically successful and safer procedure, particularly a lower rate of iatrogenic esophageal mucosal perforation and the ability to make longer incisions for the myotomy [5,6].

Thus, there is increasing interest in investigating RAHM, with very promising results. However, despite the advantages reported in the literature, the adoption of the RAHM in lieu of LHM as the gold standard for achalasia is still controversial, especially given the higher costs associated with RAHM and the lack of data proving a clear and tangible benefit compared to LHM. Although not the first in the literature, this is the largest and most comprehensive systematic review and meta-analysis of cohort studies comparing the clinical outcomes, technical success, and complication rates of RAHM and the conventional LHM.

## Review

Methodology

The present investigation was conducted with the utmost fidelity to a previously established methodology that was collectively assented to by all contributing authors of the research, in conjunction with adherence to the directives outlined in the Preferred Reporting Items for Systematic Reviews and Meta-Analyses (PRISMA) guidelines. A comprehensive search of the literature was conducted to ensure a meticulous and thorough analysis.

Literature Search Strategy

**Figure 1:**
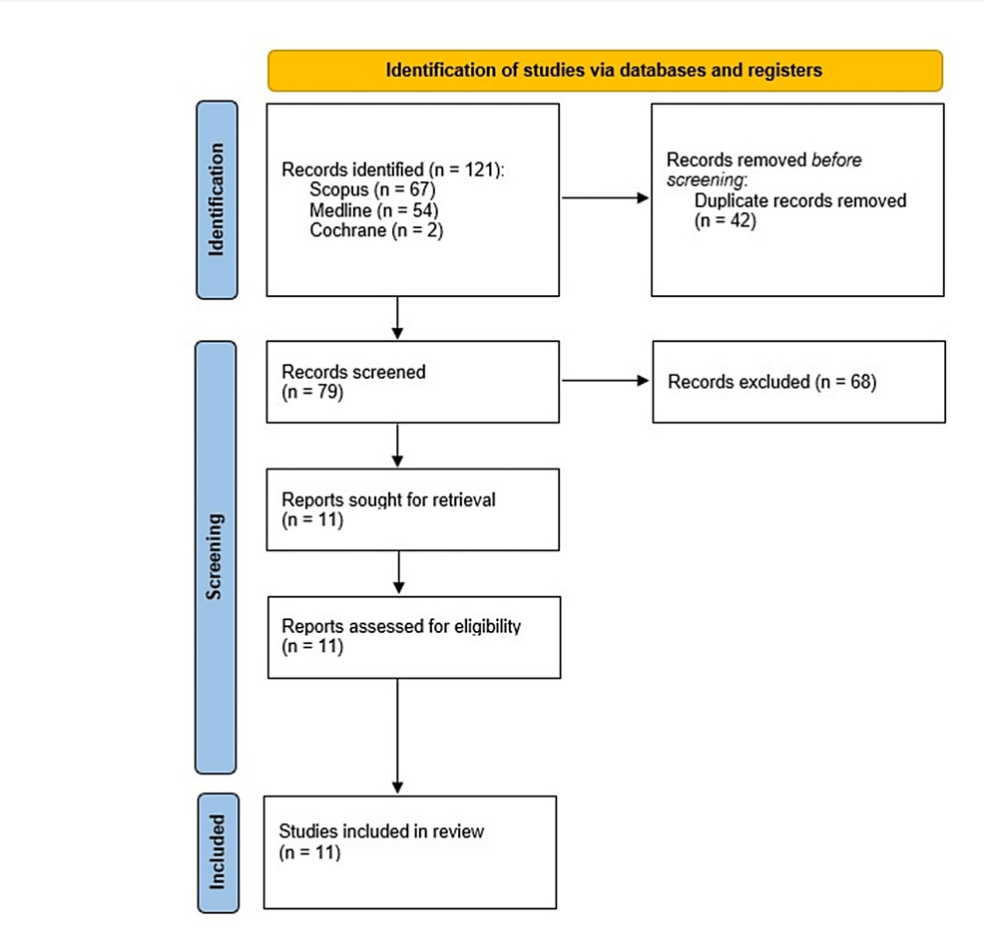
Preferred Reporting Items for Systematic Reviews and Meta-Analyses protocol.

A comprehensive literature search was performed to ensure a meticulous and thorough analysis using PubMed, Medline, and the Cochrane Central Register, We used multiple combinations of the following keywords: “robotic,” “robot,” “laparoscopic,” “Heller myotomy,” “achalasia.” Only articles published in the English language between 2000 and 2023 were included. Articles directly comparing the perioperative course, clinical outcomes, and complication rates of RAHM and LHM were selected. Data on intraoperative variables, clinical outcomes, and complications were collected and analyzed. The PRISMA flowchart (Figure 1) highlights the articles found and included in the literature review.

Data Extraction

Data pertaining to demographics such as sample size for each group, age, sex, preoperative body mass index, preoperative gastroesophageal reflux disease status, perioperative dysphagia, incidence of esophageal mucosal perforation, and overall morbidity and mortality were extracted for each included study. Two investigators, KA and AB, ensured data validity by reaching a consensus through comparison. The Cochrane Collaboration RevMan version 5.3 was employed for data analysis.

Quality and Publication Bias Evaluation

The Newcastle-Ottawa Quality Assessment Scale 7 was used as an evaluation tool to assess nonrandomized controlled trials (non-RCTs). The scale ranges from 0 to 9 stars. Studies evaluated with a score equal to or higher than 5 were considered to have adequate methodological quality and were included. There were no RCTs in the literature to be included. Two investigators (KA and AB) rated the included studies independently and a final decision was reached by consensus. The risk of publication bias was evaluated by the visual inspection of funnel plots.

Results

A total of 11 studies (Table 1) were included in this meta-analysis, comprising 3,543 patients in the RAHM subgroup and 15,434 patients in the LHM subgroup. These studies were published between 2005 and 2023 and originated from the United States, Venezuela, Switzerland, and Germany.

**Table 1:** Summary of the studies selected for the systematic review and meta-analysis. N = number of patients; Age = years (mean ± SD)

Study ID	Type of study	Journal	Country	Date Published	Patients		Female patients		Mean age	
					Robotiv	Laparoscopic	Robotic	Laparoscopic	Robotic	Laparoscopic
Sanchez et al. [7]	Case-control	Robotic Surgery	Venezuela	2011	13	18	NA	NA	38	40.7
Ali et al. [8]	Cohort	Surgical Endoscopy	USA	2019	44	40	16	18	58.25 ± 8	54.25 ± 6.25
Chacko et al. [9]	Cohort	Surgical Laparoscopy Endoscopy and Percutaneous Techniques	USA	2022	1859	9,703	997	4,834	53.2 (16.4)	54.4 (16.8)
Gass et al. [10]	Cohort	BMC Surgery	Switzerland	2022	11	32	6	9	59.55 ± 5.97	55.8 ± 4.80
Perry et al. [11]	Cohort	Surgical Endoscopy	USA	2014	56	19	28	8	47.5 ± 16.4	47.8 ± 14.0
Arcerito et al. [12]	Cohort	Society of Laparoscopic & Robotic Surgeons	USA	2022	15	96	NA	NA	49 (22–96)	49 (22–96)
Kim et al. [13]	Cohort	Robotic Surgery	USA	2019	37	35	21	15	61.7	59.5
Horgan et al. [14]	Cohort	The Society for Surgery of the Alimentary Tract	USA	2005	59	62	30	33	42 ± 19	48 ± 19
Rabe et al. [15]	Cohort	Robotic Surgery	Germany	2023	47	31	22	18	51.62 ± 4.62	51.37 ± 3.62
Siva Raja et al. [16]	Cohort	Thoracic and Cardiovascular Surgery	USA	2022	122	206	122	206	48 ± 15	46 ± 16
Huffmanm et al. [17]	Cohort	Mosby	USA	2007	24	37	14	14	57 ± 17.50	54.75 ± 15.25

Mean Operative Time

**Figure 2:**
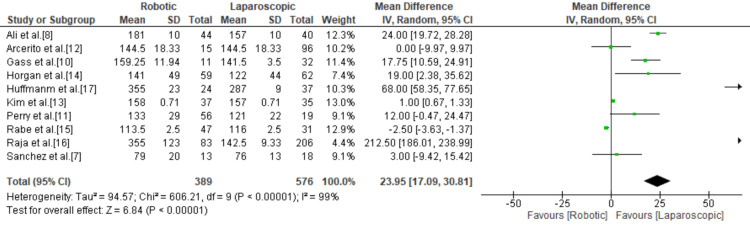
Forest plot of mean operative time. [7,8,10-17].

The operative time was significantly higher in the robotic group compared to the laparoscopic group with a total mean difference of 23.95 (95% confidence interval (Cl) = 17.09, 30.81; p < 0.00001; I^2^ = 99%) (Figure 2).

Length of Hospital Stay

**Figure 3:**
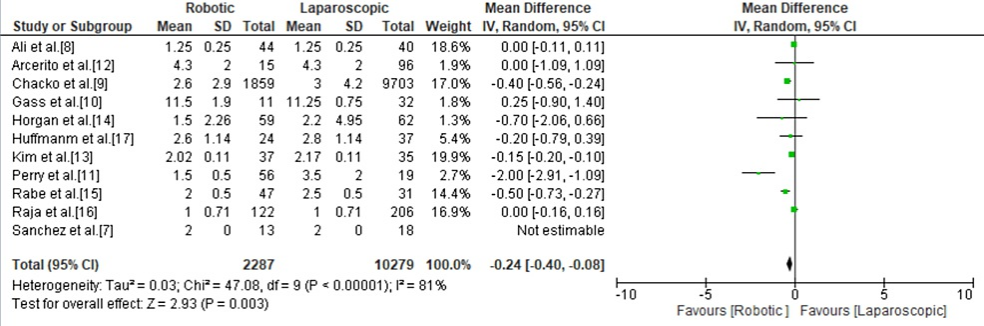
Forest plot of length of hospital stay. [7-17].

Patients in the robotic group stayed fewer days in the hospital compared to the laparoscopic group with a total mean difference of -0.24 (95% Cl = -0.40, -0.08; p < 0.00001; I^2^ = 81%) (Figure 3).

Blood Loss

**Figure 4:**
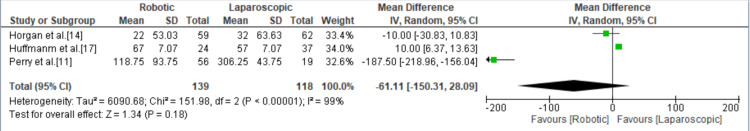
Forest plot of blood loss (in mL). [11,14,17].

Of the 11 included studies, three mentioned the estimated blood loss that was significantly lesser in the robotic group at 22-118.75 mL compared to the laparoscopic group at 32-306.25 mL with a total mean difference of -61.11 (95% CI = -150.31, 28.09; p < 0.00001; I^2^ = 99%) (Figure 4).

Conversion to Open

**Figure 5:**
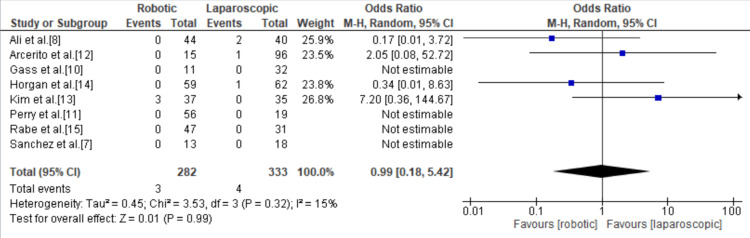
Forest plot of the odds ratio of conversion to open surgery. [7,8,10-15].

Of the 11 included studies, eight mentioned conversion to open surgery with a total of seven events with no statistically significant difference between the robotic and laparoscopic groups. The odds ratio was 0.99 (95% CI = 0.18, 5.42; p = 0.99; I^2^ = 15%) (Figure 5).

The Eckardt Symptom Score

**Figure 6:**
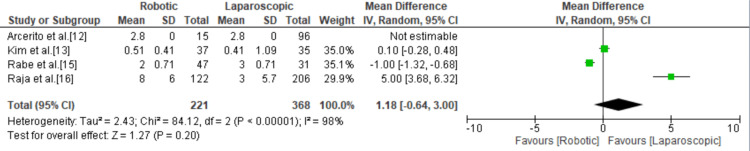
Forest plot of the mean Eckardt score. [12,13,15,16].

Only four studies mentioned the Eckardt score and showed no significant difference between the robotic and laparoscopic groups with a mean difference of 1.18 (95% CI = -0.64, 3; p = 0.20; I^2^ = 98%) (Figure 6).

Postoperative Gastroesophageal Reflux Disease

**Figure 7:**
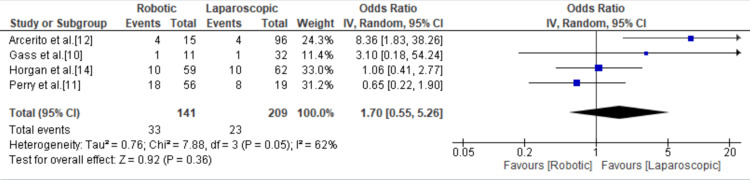
Forest plot for postoperative gastroesophageal reflux disease. [10-12,14].

Of the 11 included studies, four mentioned gastroesophageal reflux as a postoperative complication with a total of 56 events and showed no significant difference between the robotic and laparoscopic group with an odds ratio of 1.70 (95% CI = 0.55, 5.26; p = 0.36; I^2^ = 62%) (Figure 7).

Esophageal Perforation

**Figure 8:**
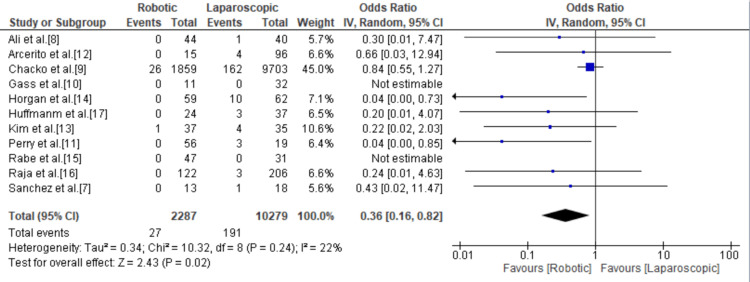
Forest plot for esophageal perforation. [7,8,10-17].

With a total of 218 events, esophageal perforation was mentioned in every study and was significantly lower in the robotic group compared to the laparoscopic group with an odds ratio of 0.36 (95% CI = 0.16, 0.82; p = 0.02; I^2^ = 22%) (Figure 8).

Recurrence

**Figure 9:**
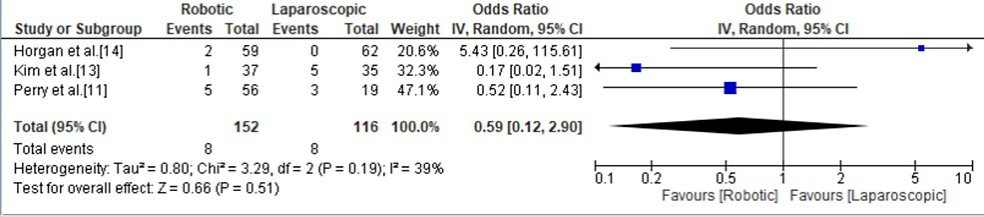
Forest plot for recurrence. [11,13,14].

The recurrence of symptoms was mentioned in three studies and occurred eight times in each group with no significant difference and an odds ratio of 0.59 (95% CI = 0.12, 2.90; p = 0.51, I^2^ = 39) (Figure 9).

Reintervention

**Figure 10:**
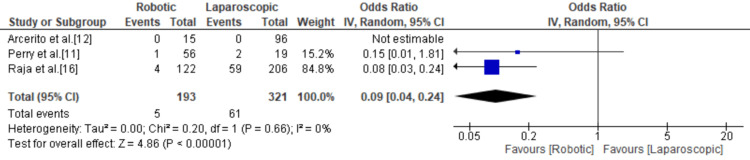
Forest plot for reintervention. [11,12,16].

Three studies mentioned reintervention with a total of 66 events with a higher rate in the robotic group compared to the laparoscopic group resulting in an odds ratio of 0.09 (95% CI = 0.04, 0.24; p < 0.00001; I^2^ = 0) (Figure 10).

Symptom Relief

**Figure 11:**
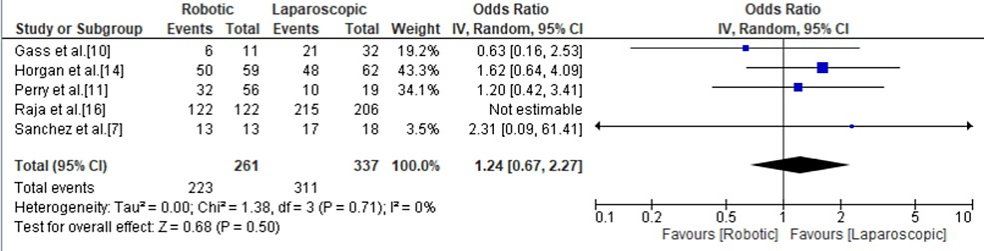
Forest plot of the odds ratio of symptom relief. [7,10,11,14,16].

Five studies focused on postoperative patient symptom relief but showed no significant difference between both techniques with an odds ratio of 1.24 (95% CI = 0.67, 2.27; p = 0.50; I^2^ = 0%) (Figure 11).

Discussion

RAHM has become an increasingly popular alternative to the gold standard laparoscopic sleeve gastrectomy since it was first described by Melvin et al. in 2001 [4]. The aim of incorporating the robotic approach into this procedure was to evolve it by overcoming the technical challenges associated with the conventional technique, namely, increasing degrees of movement, improving vision, and avoiding the fulcrum effect [18]. This was further driven after several studies showed a lower esophageal mucosal perforation rate in RAHM and faster intracorporeal suturing of Dor fundoplication compared to the conventional LHM [14,19,20], with the volume of RAHM overtaking LHM starting in the mid-2010s [16].

The operative time of conventional LHM was significantly shorter than RAHM. The mean operative time was found to be 23.95 minutes longer (95% Cl = 17.09, 30.81; p < 0.00001; I^2^ = 99%) in RAHM compared to LHM. This is most likely due to the time needed to prepare and dock the robotic system and because most studies were published at a time when authors were still on the learning curve of the robotic system. This is in conflict with previous studies that showed no statistically significant difference in operative time between RAHM and LHM, including a meta-analysis published in 2019 by Milone et al. [21]. However, this meta-analysis included a larger number of publications with more cases of RAHM and LHM. Nonetheless, we do not believe this finding will deter surgeons from adopting RAHM and expect that this gap in operative time will become insignificant and even reverse in the near future as hospital systems become efficient with the robotic system and surgeons become more experienced and comfortable with the procedure. This change is already being reported in some of the more recent literature, such as Raja et al. in 2022, which found RAHM to be slightly faster than LHM in their study [16].

Intraoperative blood loss was found to be significantly less in the robotic group compared to the laparoscopic group. The volume of blood loss documented for RAHM was 22-118.75 mL versus 32-306.25 mL for LHM, with a total mean difference of -61.11 (95% CI = -150.31, 28.09; p < 0.00001; I^2^ = 99%) in favor of RAHM. In addition, RAHM was associated with a significantly shorter hospital stay, with a mean hospital stay of 3.17 days for RAHM versus 3.27 days for LHM, with a mean difference of -0.24 (95% Cl = -0.40, -0.08; p < 0.00001; I^2^ = 81%) in favor of RAHM. There were seven instances of conversion to open surgery, three instances in the robotic group and four instances in the laparoscopic group, with no statistically significant difference in the risk of conversion to open with RAHM and LHM with an odds ratio of 0.99 (95% CI = 0.18, 5.42; p = 0.99; I^2^ = 15%). The meta-analysis by Milone et al. in 2019 showed there was no statistically significant difference in the length of hospital stay, intraoperative bleeding, and conversion rates between RAHM and LHM [21]. Thus, our findings show an improvement in the intraoperative and perioperative course of RAHM as it continues to evolve and become more widely adopted.

Esophageal mucosal perforation is a relatively common but potentially devastating early complication of achalasia surgery. A systematic review by Maurice et al. in 2018 reported a mucosal perforation rate of 7.7% associated with LHM [22]. In this meta-analysis, the mucosal perforation rate was 1.18% in RAHM versus 1.86% in LHM. We found that RAHM was associated with a significantly lower risk of esophageal perforation compared to LHM with an odds ratio of 0.36 (95% CI = 0.16, 0.82; p = 0.02; I^2^ = 22%). Ballouhey et al. attributed this important finding to the fact that laparoscopic instruments are rigid and without any degree of freedom, such that the esophageal mucosa can get excessively retracted during the myotomy and thus is at higher risk of perforation. This is especially true during gastric dissection, where it is very difficult to find the plane between the muscle wall and the mucosa. On the other hand, the robotic arm, with its ability to use articulating instruments with seven degrees of freedom, allows for the tangential dissection of the muscular layer without applying too much pressure on the mucosa [23]. In addition, the three-dimensional image produced by the robot grants the surgeon a better sense of depth [14]. Our findings are in line with the literature, including Milone et al.’s meta-analysis published in 2019, which confirmed that RAHM was associated with a significantly lower intraoperative esophageal perforation rate.

There was no significant difference in the reported relief and recurrence of symptoms between RAHM and LHM. While LHM seemed to have a higher rate of symptom relief with an odds ratio of 1.24 (95% CI = 0.67, 2.27; p = 0.50; I^2^ = 0%), RAHM was found to have a lower rate of symptom recurrence with an odds ratio of 0.59 (95% CI = 0.12, 2.90; p = 0.51; I^2^ = 39). However, neither of these differences reached statistical significance. Given that recurrence rates following LHM can reach 10% [24,25], our findings suggest that RAHM may be associated with a higher long-term success rate when compared to LHM. Of interest, there was a significantly higher rate of reintervention in RAHM compared to LHM.

A major limitation of this review is the lack of data comparing the costs of RAHM and LHM. Shaligram et al. highlighted the fact that RAHM was associated with higher costs compared to LHM [26]. This was a point of contention, especially as the literature had shown equivalency between LHM and RAHM in terms of mortality, morbidity, and length of hospital stay. While this meta-analysis confirms some of these findings, namely, no statistically significant difference in morbidity and mortality between LHM and RAHM, it also reports that RAHM is associated with a significantly lower risk of intraoperative esophageal perforation, significantly lower symptom recurrence, significantly smaller estimated blood loss, and a significantly shorter length of hospital stay. Thus, we are of the opinion that the robotic approach is becoming ever more justifiable as experience, volume, and efficiency improve.

## Conclusions

RAHM is associated with a lower esophageal perforation rate, less blood loss, shorter hospital stay, and a similar overall 30-day complication rate compared to LHM. However, whether these findings translate to improved long-term morbidity and mortality and whether they can justify the higher costs associated with incorporating the robotic system remains to be seen and we do not recommend drawing any major conclusions on these matters from this article. Further longitudinal RCTs directly comparing RAHM with LHM, with an emphasis on the associated costs of each procedure, are needed to better elucidate the long-term outcomes and financial feasibility of RAHM.
